# Post-Operative Plasma Osteopontin Predicts Distant Metastasis in Human Colorectal Cancer

**DOI:** 10.1371/journal.pone.0126219

**Published:** 2015-05-11

**Authors:** Lui Ng, Timothy Ming-Hun Wan, Colin Siu-Chi Lam, Ariel Ka-Man Chow, Sunny Kit-Man Wong, Johnny Hon-Wai Man, Hung-Sing Li, Nathan Shiu-Man Cheng, Ryan Chung-Hei Pak, Alvin Ho-Kwan Cheung, Thomas Chung-Cheung Yau, Oswens Siu-Hung Lo, Dominic Chi-Chung Foo, Jensen Tung-Chung Poon, Ronnie Tung-Ping Poon, Roberta Wen-Chi Pang, Wai-Lun Law

**Affiliations:** 1 Department of Surgery, Li Ka Shing Faculty of Medicine, The University of Hong Kong, Hong Kong SAR, China; 2 Centre for Cancer Research, Li Ka Shing Faculty of Medicine, The University of Hong Kong, Hong Kong SAR, China; University of Alabama at Birmingham, UNITED STATES

## Abstract

**Background:**

The overall prognosis of colorectal cancer (CRC) patients is unsatisfactory due to cancer metastasis after operation. This study aims to investigate the clinical significance of plasma osteopontin (OPN) levels as minimally invasive, predictive, and surrogate biomarkers for prognosis of CRC patients.

**Methods:**

This randomized study design consists of pre-operative and post-operative plasma samples from a total of 79 patients. We determined plasma levels of OPN by ELISA and examined their correlation with the clinicopathological parameters of CRC patients. The effects of endogenous and exogenous OPN on CRC metastasis were investigated by examination of the effect on regulators of epithelial to messenchymal transition and migration assay.

**Results:**

Our findings demonstrated for the first time the clinical correlation of plasma OPN with metastasis of CRC patients. High post-operative plasma OPN level (>153.02 ng/ml) associated with development of metastasis after curative resection (p<0.001). Moreover, post-operative plasma OPN level correlated with disease-free survival of CRC patients (p=0.009) and was an independent factor for predicting development of metastasis in CRC patients after curative resection (p=0.036). Our *in vitro* model showed that OPN ectopic expression induced DLD1 cell migration through Snail and Twist1 overexpression and E-cadherin repression, and secretory OPN level enhanced cell migration.

**Conclusions:**

The results of the current study suggest that post-operative plasma OPN correlated with post-operative metastasis, suggesting that it is a potential non-invasive biomarker for the development of future metastasis in CRC patients. In addition, OPN was shown to be involved in the metastatic process and thus inhibition of OPN is a potential therapeutic approach to treat CRC patients.

## Introduction

Colorectal cancer (CRC) is the third most common malignancy around the world [1]. Annually, over 1.2 million people develop CRC globally, with more than 600,000 patients die from the disease in 2008 [2]. Incidence and mortality rates for CRC have declined as a result of improved tests that allow early detection of the cancer, when it can be more easily treated by surgery and chemotherapy along with radiotherapy [3]. Despite those advances in clinical treatment, the overall prognosis of CRC patients is still unsatisfactory due to cancer metastasis. Therefore, it is important to develop additional biomarkers in order to enhance the prognosis of CRC patients by prediction or early detection of occult metastasis.

Blood-based, minimally invasive markers would be increasing important in CRC screening and monitoring of CRC patients. Carcinoembryonic antigen (CEA) and CA19-9 have been commonly assessed in CRC patients, but with varying results depending on the study design and the study population [4], and their clinical association with cancer metastasis have not been demonstrated thus far. Although numerous biomarkers are under evaluation for the detection of CRC from serum, none of them has sufficient sensitivity and specificity to be considered in the current guidelines [4].

Osteopontin (OPN) is a secreted glycoprotein with a multi-domain structure and functions characteristic of an extracellular protein [5]. It is a small integrin-binding ligand *N*-linked glycoprotein (SIBLING) that binds to cell surface receptors including integrins and CD44 [6]. OPN is expressed in many tissues and secreted into body fluids, including blood, milk and urine [7–9], which has important physiological roles in bone remodeling, immune response and inflammation [10]. It is also a tumor-associated protein and elevated OPN levels are associated with cellular proliferation, invasion and angiogenesis via altered activity of matrix metalloproteinases, the epidermal growth factor receptor and PI3K-AKT signaling [11, 12]. These preclinical studies suggest that plasma OPN could be an important non-invasive biomarker of occult systemic metastases. In accordance, in a recent meta-analysis of over 228 publications, high tumor or plasma OPN levels correlated with decreased disease-free survival and overall survival in multiple tumor types, including lung cancer, breast cancer, prostate cancer, head and neck cancer and liver cancer [13].

Increased levels of OPN mRNA and protein have been demonstrated in CRC comparing to the non-tumor tissue [14–17], and the high expression correlated with metastatic features such as lymph node metastasis and distant metastasis [17]. However, Nitsche *et al*’s study which investigated more than 200 patients with stage II colon cancer demonstrated that tumor tissue level of osteopontin was useful for detecting the presence of colon cancer, but not for predicting the prognosis of the patients [18]. These studies suggested that OPN tissue level was not yet confirmative to detect or predict CRC metastasis. Blood OPN level has been suggested as a potential detective biomarker of CRC, yet its clinical correlation with CRC metastasis has not been demonstrated thus far. In addition, most of the cancer studies focused on the clinical significance of pre-operative plasma OPN level, while the post-operative level in cancer patients has seldom been investigated. Therefore, in this study, we will examine the clinicopathogical significance as well as the prognostic potential of pre-operative and post-operative plasma OPN levels of CRC patients.

## Materials and Methods

### Patients and specimens

The human sample collection protocol has been approved by the Institutional Review Board (IRB) of the University of Hong Kong, and all clinical investigation has been conducted according to the principles expressed in the Declaration of Helsinki. Informed written consent has been obtained from the participants. Pre-operative blood samples were obtained from 96 patients who underwent surgical resection of primary CRC at the Department of Surgery, Queen Mary Hospital, The University of Hong Kong, between 1998 and 2007. Tissue samples were obtained from 32 out of the 96 patients, immediately frozen in liquid nitrogen and kept at -80°C until analysis. The post-operative blood was obtained from patients during their post-operative follow-up visit to our clinic (usually within 6 months after surgical operation). Blood was anticoagulated by EDTA and then centrifuged at 2,500 rpm for 10 min. The plasma was collected, aliquoted, and snap frozen at -80°C till use. Clinicopathological data and blood CEA level were obtained from the patient database of our hospital.

### Enzyme-linked immunosorbent assay

Plasma level of OPN or secretory OPN level in CRC cell-lines were measured using a commercial enzyme-linked immunosorbent assay kit (R&D Systems, Inc., Minneapolis, MN) and 100 μl of diluted (1:125) plasma samples/200 μl of diluted cell-culture media. The plates were firstly coated with OPN capture antibody overnight. After three washes with PBS-0.5% tween 20, the plates were blocked with 1% filtered BSA in PBS for 1 hour. 0.1 mL of each sample was added to the plate and incubated for 2 hours at room temperature, followed by addition of 100 μl OPN biotinylated antibody and incubated for 2 hours at room temperature. After three washes, 100 μl of horseradish-conjugated strepavidin solution was added and incubated for 20 minutes at room temperature. Finally, 100 μl of tetramethylbenzidine substrate solution was added and incubated for 5 minutes in the dark, followed by the addition of 50 μl of stop solution. Absorbance was measured at 450 nm with wavelength correction at 570 nm.

### RNA extraction and DNase I treatment

Total RNA was extracted using Trizol reagent and Purelink RNA mini kit (Life Technologies, Carlsbad, CA). Briefly, 50–100 mg of frozen tissue sample was homogenized in 1 ml Trizol Reagent using a homogenizer, whereas cells adherent on culture dish were directly lysed by adding 1 ml of Trizol Reagent, pipetting the lysates several times, and transferring to a sterile 1.5 ml centrifuge tube. Following 5 min of incubation at room temperature, 0.2 ml chloroform per 1 ml Trizol Reagent used was added and the tube was shaken vigorously by hand for 15 sec. Following 3 min-incubation, the sample was centrifuged at 12,000 x g for 15 min. The colorless upper aqueous phase was transferred to a new sterile tube and equal volume of 70% ethanol was added and the mixture was mixed well. The sample was transferred to a spin cartridge and centrifuged at 12,000 x g for 15 sec at room temperature, followed by washing step with 700 μl Wash Buffer I once and 500 μl Wash Buffer II twice. The spin cartridge was dried by centrifugation at 12,000 x g for 1 min. 30 μl RNased-Free water was added to the spin cartridge, incubated for 1 min and RNA was eluted in a new tube by centrifugation at 12,000 x g for 2 min. The RNA was then proceeded to DNase I digestion by adding 3 μl 10X DNase I buffer and 3 μl DNased I, Amplification Grade (Life technologies) to 24 μl RNA sample. Following 15 min-incubation at room temperature, RNA was purified using Purelink RNA mini kit (Life Technologies). Briefly, 1 volume of freshly prepared lysis buffer with 2–mercaptoethanol (10 μl per 1 ml lysis buffer) and one volume of absolute ethanol was added to each RNA sample and mixed well. The sample was transferred to a spin cartridge and centrifuged at 12,000 x g for 15 sec at room temperature, followed by washing step with 700 μl Wash Buffer I once and 500 μl Wash Buffer II twice. The spin cartridge was dried by centrifugation at 12,000 x g for 1 min. 30 μl RNased-Free water was added to the spin cartridge, incubated for 1 min and RNA was eluted in a new tube by centrifugation at 12,000 x g for 2 min. The RNA yield and quality were analyzed by NanoDrop 2000 (Thermo Scientific).

### cDNA synthesis and quantitative real-time polymerase chain reaction

2.0 μg total RNA was reverse-transcribed with SuperScriptII RT-PCR kit (Invitrogen, Carlsbad, CA) in accordance with the instructions of the manufacturer. Real-time PCR was performed in a final volume of 15 μl containing 1.5 μl RT transcript, 0.2 μM of each primer, 1X ROX reference dye and 7.5 μl of FastStart Universal SYBR Green Master (ROX) (Roche Diagnostics, Switzerland, Basel). A no RT transcript control was included for each gene to ensure the signal was truly driven by target gene amplification. The primer sequences were as follows: OPN-Forward Primer: 5’-TGGGGGTCACTGCAATTAG-3, OPN-Reverse Primer: ‘5’-TGGGGCTAGGAGATTCTG-3’; E-cadherin-Forward Primer: 5’- TGGAGGAATTCTTGCTTTGC-3’; E-cadherin-Reverse Primer: 5’- CGTACATGTCAGCCAGCTT-3’; Snail-Forward Primer: 5’-CAGACCCACTCAGATGTCAA-3’, Snail-Reverse Primer: 5’-CATAGTTAGTCACACCTCGT-3’; Twist1-Forward Primer: 5’- GGGAGTCCGCAGTCTTAC-3’, Twist1-Reverse Primer: 5’- CCTGTCTCGCTTTCTC-TTT-3’; GAPDH- Forward Primer: 5’- GTCTCCTCTGACTTCAACAGCG -3’, GAPDH- Reverse Primer: 5’- ACCACCCTGTTGCTGTAGCCAA -3’. Real-time PCR was carried out using the ABI 7900HT Fast Real-Time PCR System (Applied Biosystems, Foster, CA) at 95°C for 10 min, followed by 40 cycles at 95°C for 15 sec and at 56°C for 1 min. Each assay was done in triplicate, the average was calculated and the expression level of target mRNA was expressed as fold to expression of GAPDH. For cell-line experiments, fold-change was calculated based on the change in abundance of transcript of interest (normalized to GAPDH) from experimental group compared to the abundance of transcript of interest (normalized to GAPDH) from control group.

### Plasmid construction

OPN full-length coding sequence was amplified by OPN-*Kpn* I-Forward Primer CGGGGTACCGTGCCAGCCAAACAACAAA and OPN-Xba I-Reverse Primer TGCTCTAGATGCAAAGTGAGAAATTGTATTT, using Platinum *Taq* DNA Polymerase High Fidelity (Life Technoloies) according to the manufacturer’s instruction. The PCR cycle was 94°C for 2 min, followed by 30 cycles of 94°C for 30 sec, 56°C for 30 sec and 68°C for 2 min. The OPN PCR product was purified by Qiagen PCR purification system (Valencia, CA, USA) according to the manufacturer’s protocol. The pcDNA3.1 vector (Invitrogen) and OPN purified PCR products were digested by *Kpn*I and *Xba*I restriction enzymes (New England Biolabs), purified and ligated by DNA ligase (New England Biolabs). The ligation product was transformed into DH5α competent cells (Life Technologies) according to the manufacturer’s instructions. The plasmids were extracted by the Qiaprep Spin Miniprep Kit (Qiagen) sequence fidelity was confirmed by DNA sequencing.

### Cell-lines, tissue culture, transfections and reagents

CRC cell-lines were maintained in DMEM with 10% fetal bovine serum and antibiotics (Invitrogen, Carlsbad, CA), 5% CO_2_ at 37°C. DLD1 and SW480 cells were transiently transfected with vector control or OPN expression plasmid using Lipofectamine 2000 reagent (Invitrogen) according to the manufacturer’s instructions. DLD1-OPN1 and vector control stable clones were generated by selection in culture medium containing 1 mg/ml G418 (Roche) for 3 weeks. HCT116 and SW620 cells were transiently transfected with siRNA negative control or OPN siRNA oligonucleotides (Invitrogen) using Lipofectamine 3000 reagent (Invitrogen) according to the manufacturer’s instructions.

### Protein extraction and western blot analysis

Cell-lines were harvested, collected and lysed in RIPA buffer (Cell Signaling Technology, Danvers, MA) containing 1 mmol/L phenoylmethylsulfonyl fluoride for 1 hour on ice. The lysate was centrifuge at 12,000 x g for 15 min and supernatant was transferred to a new sterile tube. The protein concentration was determined using BCA Protein Assay Kit (Thermo Scientific). Lysed protein was suspended in sodium dodecyl sulfate sample buffer, boiled, resolved in sodium dodecyl sulfate—polyacrylamide gel electrophoresis, and transferred to PVDF membranes (GE Healthcare, Piscataway, NJ). Antibodies against E-cadherin and GAPDH (for normalization) were purchased from Cell Signaling Technology (Danvers, MA). Representative Western blots of experiments that were repeated 3 times are shown. Densitonmetry was performed on the western blots using ImageJ software.

### *In vitro* migration and invasion assay

Cells were harvested, resuspended in serum-free medium and seeded into transwell chamber (Corning or BD Biosciences). Two hundred and fifty microliters of a 50000 cell suspension in serum-free medium were added to the upper chamber which was placed into a 24-well plate with the bottom filled with 750 μl complete medium as a chemoattractant. After 3 days the number of cells migrated was determined according to the manufacturer’s instructions. Each experiment was done in duplicate, and results were presented as the mean ± SD of three independent experiments.

### Statistical analysis

The association of plasma OPN levels with continuous variables was tested with Pearson’s correlation. Student’s t-test was applied to compare difference between two groups. Differences in recurrence rates in different groups of patients with CRC were evaluated by the fisher exact test. Disease-free survival was analyzed using the Kaplan—Meier product limit method and log-rank test. Univariate and multivariate analyses in a Cox proportional-hazards model for prognostic predictors were performed. All of these statistical analyses were performed with SigmaPlot 10.0 (Systat Software Inc., San Jose, CA, USA) or SPSS 10.0 software (SPSS, Inc., Chicago, IL, USA). Statistical significance was set at p ≤ 0.05.

## Results

### Comparison of plasma OPN level between normal donors and CRC patients

We first determined the plasma OPN level from 56 normal donors and 83 CRC patients before surgical resection of their tumors. The mean plasma OPN level of CRC patients was 160.31 ng/ml, which was significantly higher than that of normal donors (115.27 ng/ml; p<0.001). We further divided the patients into early stage (stage I to II, n = 45) and advance stage (stage III to IV, n = 38). The mean plasma OPN level of advance stage patients (187.91 ng/ml) was significantly higher than that of normal donors (p<0.001), whereas level of early stage patients (137.00 ng/ml) was insignificantly higher (p = 0.052). These results suggested that plasma OPN could be a non-invasive biomarker for screening CRC patients with advance tumor stage, but its sensitivity decreased in screening early stage CRC patients.

### Correlation of pre-operative plasma OPN level with OPN transcript expression and clinicopathological parameters

**Fig 1:**
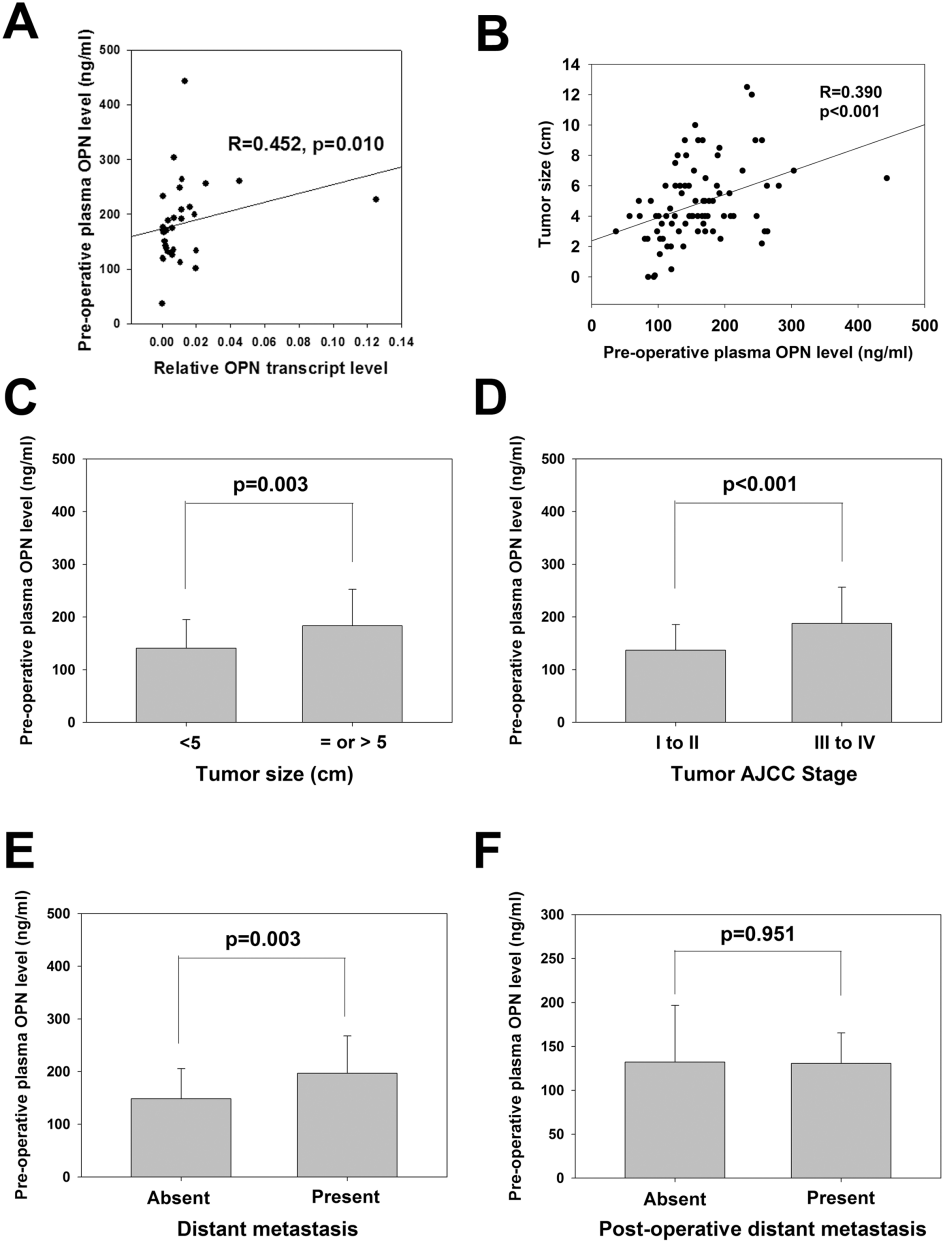
Clinicopathological significances of pre-operative plasma OPN level. (*A*) Correlation of pre-operative plasma OPN level with the paired tumor OPN transcript level in 32 CRC patients (R = 0.452, p = 0.010; Pearson correlation). (*B*) Correlation of pre-operative plasma OPN level with tumor size (R = 0.390, p<0.001; Pearson correlation). (*C*) Comparison of pre-operative plasma OPN levels in patients with tumor ≥5 or <5 (p = 0.003; Student’s t-test) (left). (*D*) Comparison of pre-operative plasma OPN levels in patients with lower (I to II) or higher stage (III to IV) (p<0.001; Student’s t-test). (*E*) Comparison of pre-operative plasma OPN levels in patients with or without distant metastasis (p = 0.003; Student’s t-test). (*F*) Comparison of pre-operative plasma OPN levels in patients with or without post-operative metastasis (p = 0.951; Student’s t-test). All data are representative of three independent experiments.

We next examined the correlation between the pre-operative plasma OPN level and the paired tumor OPN transcript level in 32 CRC patients. As shown in Fig 1A, there was a positive correlation between the OPN level in plasma and CRC tumor (R = 0.452, p = 0.0010), suggesting that plasma OPN level in CRC patients was indicative of their CRC OPN expression. We then examined the correlation of pre-operative plasma OPN level of 83 CRC patients with their clinicopathological data (Table 1). Plasma OPN level did not correlate with age, gender and lymph node metastasis. Significantly higher plasma OPN level was detected in patients with tumor size over 5 cm (180.4 vs 140.7 ng/ml, p = 0.003; 1C), higher stage (187.9 vs 137.0 ng/ml, p<0.001; Fig 1D) and metastatic CRC (197.0 vs 148.7 ng/ml, p = 0.003; Fig 1E). The pre-operative plasma OPN level also positively correlated with tumor size (R = 0.390, p<0.001; Fig 1B). These results revealed that pre-operative plasma OPN was a potential non-invasive biomarker for tumor progression and metastasis. We also examined whether pre-operative plasma OPN level correlated with future metastasis of CRC patients who showed no distant metastasis at operation. Our results showed that such level showed no significant difference between patients with and without future metastasis (132.09 vs 130.63 ng/ml, p = 0.951; Fig 1F), indicating that pre-operative plasma OPN level was not prognostic for future metastasis.

**Table 1:** Clinicopatholgical Correlation of Pre-operative Plasma OPN level (N = 83).

Clinicopathological features	Category	Number of cases*	Pre-operative plasma OPN level (ng/ml) (Mean ± SEM)	p value
Age	<65	32	153.5 ± 10.17	0.446
	> or = 65	51	164.6 ± 9.45	
Gender	Male	58	155.7 ± 7.40	0.319
	Female	25	171.0 ± 15.71	
Tumor Size	< 5 cm	44	140.7 ± 8.23	0.003
	≥ 5 cm	36	180.4 ± 11.52	
Lymph node metastasis	Absent	37	158.1 ± 11.29	0.618
	Present	41	165.5 ± 9.51	
Tumor AJCC stage	I to II	45	137.0 ± 7.27	0.001
	III to IV	38	187.9 ± 11.16	
Distant metastasis	Absent	63	148.7 ± 7.20	0.003
	Present	20	197.0 ± 15.80	

*In some category, the total number of cases is less than 83 due to incomplete information.

### Clinical significance of post-operative plasma OPN levels

**Fig 2:**
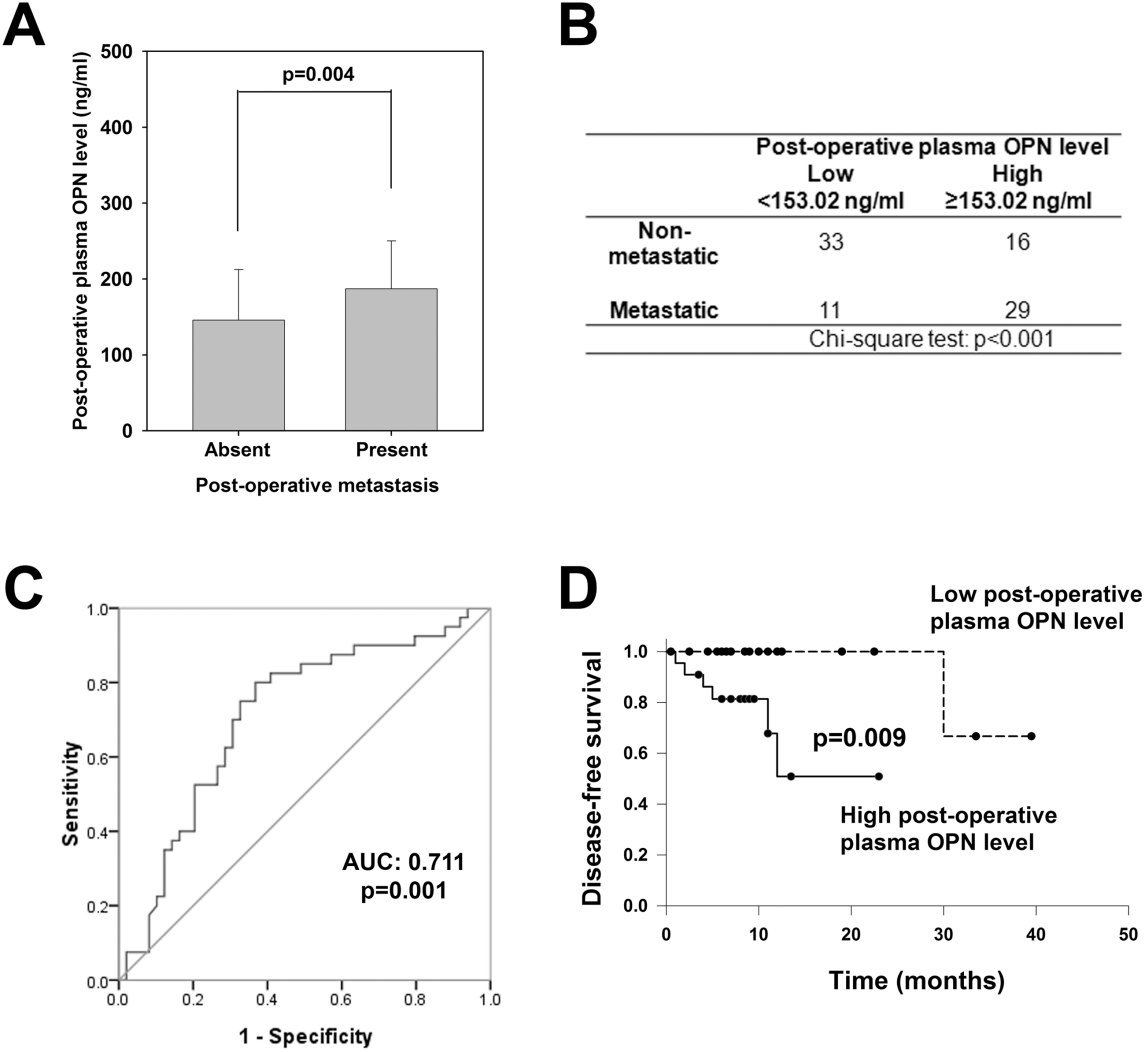
Clinicopathological significances of post-operative plasma OPN level. (*A*) Comparison of post-operative plasma OPN levels in patients with or without post-operative metastasis (p = 0.004; Student’s t-test). (*B*) Incidence rates of post-operative metastasis in CRC patients with high (≥153.02 ng/ml) or low (<153.02 ng/ml) post-operative plasma OPN level. (C) ROC curve using post-operative plasma OPN level for discriminating patients with post-operative metastasis from those without post-operative metastasis. (D) Correlation of post-operative plasma OPN with disease-free survival of CRC patients (p = 0.009; Log-rank test). All data are representative of three independent experiments.

We next studied the prognostic value of post-operative plasma OPN level for the development of post-operative metastasis in another cohort of 89 CRC patients. 40 of them developed post-operative metastasis within 30 months, and these patients displayed significantly higher post-operative plasma OPN level than the 49 non-metastatic patients (187.0 vs 145.7 ng/ml, p = 0.004; Fig 2A). Applying the overall median post-operative plasma OPN level (153.02 ng/ml) of CRC patients in this study as the threshold value, CRC patients with high post-operative plasma OPN level were more likely to develop distant metastasis (29 out of the 45 patients) than those with low post-operative plasma OPN level (11 out of 44, p<0.01; Fig 2B). To illustrate the performance of the post-operative plasma OPN as prediction biomarker, ROC curve was constructed. The area under curve (AUC) of the ROC curve was 0.711 (Fig 2C, 95% CI: 0.601 to 0.821), and the sensitivity and specificity were 0.700 and 0.694, respectively.

### Comparison of prognostic significance between plasma OPN and blood CEA

To confirm that post-operative plasma OPN level was an important prognostic factor for future metastasis, we examined the prognostic values of post-operative plasma OPN level and pre-operative plasma OPN level for disease-free survival (DFS) of 44 stage I to III CRC patients with thorough operation and follow-up information. Stage IV CRC patients where distal metastasis can be present before surgery were excluded from this analysis. As shown in Fig 2D, CRC patients with lower post-operative plasma OPN level showed a significantly longer DFS than those over the threshold level (p = 0.009), whereas pre-operative plasma OPN level did not correlate with DFS of CRC patients (S1 Fig). We also examined the prognostic value of CEA levels (S1 Fig). Neither pre-operative nor post-operative CEA correlated with DFS (p = 0.235 and 0.410, respectively).

### Risk factors for disease-free survival of CRC patients

We applied univariate and multivariate analyses to determine the risk factors for DFS of the above 44 CRC patients (Table 2). Among the clinical factors examined in the univariate analysis, only post-operative plasma OPN level was significantly correlated with DFS. In addition, our multivariate analysis showed that post-operative OPN level and lymph node metastasis were risk factors for DFS.

**Table 2:** Univariate and multivariate analyses of prognostic factors for disease-free survival of CRC patients.

Univariate analysis	Relative risk (95% confidence interval)	p-value
Age, year (<65 versus ≥65)	0.766 (0.090–6.497)	0.807
Gender (female versus male)	0.255 (0.049–1.340)	0.107
Tumor Size (≥ 5 versus< 5)	1.635 (0.408–6.554)	0.488
Tumor stage (III versus I to II)	3.054 (0.609–15.310)	0.175
Lymph node metastasis (yes versus no)	1.305 (0.324–5.256)	0.708
Pre-operative CEA level (high versus low)	2.960 (0.594–14.765)	0.186
Post-operative CEA level (high versus low)	1.466 (0.347–6.194)	0.603
Pre-operative OPN level (high versus low)	2.167 (0.513–9.158)	0.293
Post-operative OPN level (high versus low)	14.656 (1.748–122.866)	* 0.013
**Multivariate analysis**	**Relative risk (95% confidence interval)**	**p-value**
Age, year (≥ 65 versus< 65)	NA	
Gender (female versus male)	NA	
Tumor Size (≥ 5 versus< 5)	NA	
Tumor stage (III to IV versus I to II)	NA	
Lymph node metastasis (yes versus no)	13.367 (1.017–175.646)	*0.045
Pre-operative CEA level (high versus low)	NA	
Post-operative CEA level (high versus low)	NA	
Pre-operative OPN level (high versus low)	NA	
Post-operative OPN level (high versus low)	191.091 (1.401–2.606E4)	*0.036

Note:

* indicates the difference is statistically significant

### In vitro effect of elevated OPN transcript and secretory OPN levels

**Fig 3:**
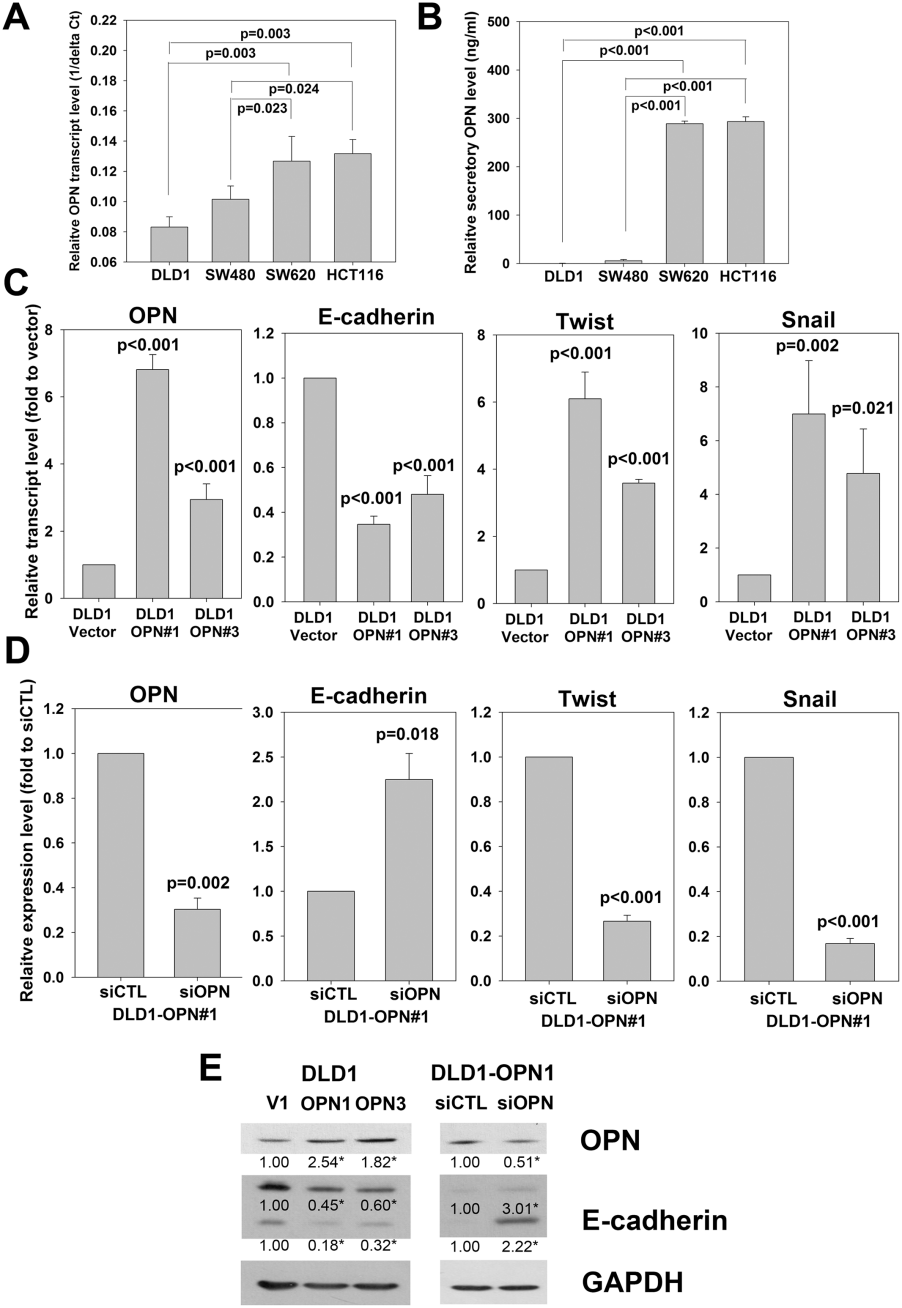
Molecular effects of OPN on EMT regulators. (*A and B*) Comparison of (*A*) transcriptional and (B) secretory OPN levels of two weakly metastatic CRC cell-lines (DLD1 and SW480) and two metastatic CRC cell-lines (SW620 and HCT116) (One way ANOVA). *(C)* OPN mRNA expression of two DLD1-OPN stable clones (DLD1-OPN#1 and #3) and vector control (DLD1-Vector) and effect of stable OPN overexpression on mRNA expression of EMT regulators E-cadherin, Twist and Snail (One way ANOVA). *(D)* OPN mRNA expression of siCTL or siOPN transfected DLD1-OPN#1 cells and effect of OPN siRNA on mRNA expression of EMT regulators E-cadherin, Twist and Snail (Student’s t-test). *(E)* Effect of OPN overexpression or siRNA on E-cadherin protein expression. GAPDH served as loading control. Densitometry was performed on the western blots using the ImageJ software and an asterisk indicates the difference was statistically significant (p<0.05) when compared with the control (DLD1-V1 or DLD1-OPN1 siCTL; One way ANOVA or Student’s t-test). All data are representative of three independent experiments.

**Fig 4:**
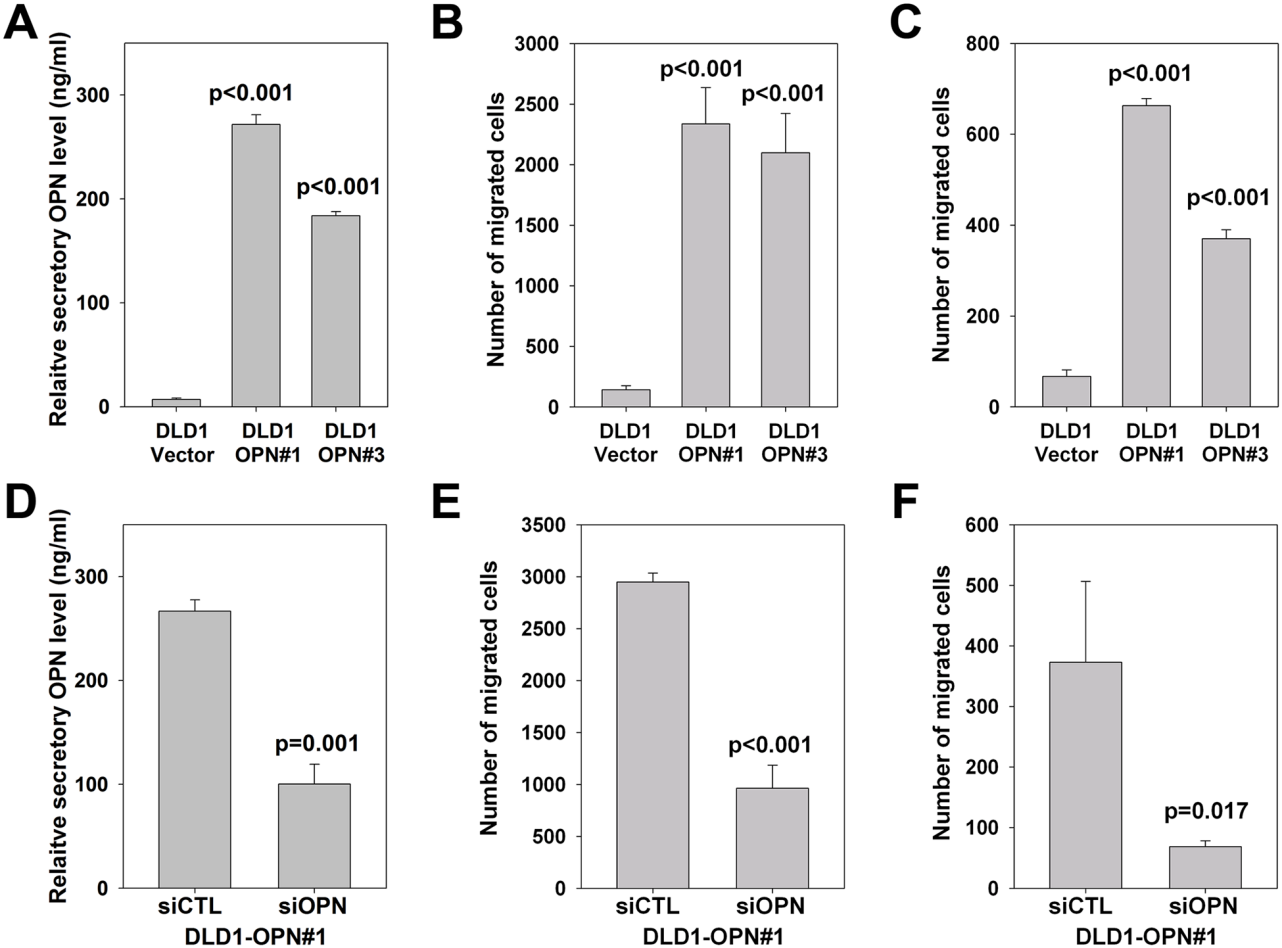
Functional effects of OPN on CRC cell migration. (*A*) Comparison of relative secretory OPN levels of two DLD1-OPN stable clones (DLD1-OPN#1 and #3) with that of DLD1-vector control (One way ANOVA). (*B*) Comparison of number of DLD1-OPN stable clones (DLD1-OPN#1 and #3) cells migrated with that of DLD1-vector control cells (One way ANOVA). (*C*) Comparison of number of DLD1 cells migrated using culture medium from DLD1-OPN stable clones (DLD1-OPN#1 and #3) or DLD1-vector control as chemoattractant (One way ANOVA). (*D*) Comparison of relative secretory OPN levels of DLD1-OPN#1 stable clone transfected with siRNA control (siCTL) or OPN siRNA (siOPN) (Student’s t-test). (*E*) Effect of siCTL or OPN siRNA transfection on migration of DLD1-OPN#1 stable cells (Student’s t-test). (*F*) Comparison of number of DLD1 cells migrated using culture medium from DLD1-OPN stable clones transfected with siCTL or siOPN as chemoattractant (Student’s t-test). All data are representative of three independent experiments.

Finally, we investigated the *in vitro* effects of OPN overexpression on secretory OPN level and metastatic feature of CRC cells. We first compared the transcriptional and secretory OPN levels of two relatively non-metastatic cell-lines DLD1 and SW480, and two metastatic cell-lines SW620 and HCT116 [19, 20]. After 3 days, both levels were significantly lower for DLD1 and SW480 cells when compared with SW620 and HCT116 cells (Fig 3A and 3B), suggesting that OPN transcript level correlated with its secretory level, and high OPN level correlated with the metastatic potential of CRC cells. We next stably overexpressed OPN in DLD1 cells and generated two stable OPN transfectants (DLD1-OPN#1 and DLD1-OPN#3), both expressed higher level of OPN transcript, protein and secretory protein than the DLD1-vector control (Figs 3C, 3E and 4A). We determined the effect on proliferation, cell invasion and cell migration. Our results showed that OPN overexpression significantly induced cell migration in DLD1 cells (Fig 4B), but did not alter the growth rate and cell invasion ability (S2 Fig). To confirm the effect of OPN on cell migration, we transiently knock-down OPN expression in DLD1-OPN stable clone by siRNA technique. Comparing to siRNA control transfected cells, DLD1-OPN cells transfected with siOPN displayed significantly lower level of OPN transcript and protein as well as secretory OPN protein (Figs 3D, 3E and 4D). More importantly, the migration rate was also significantly repressed following siOPN transfection (Fig 4E), strengthening the regulatory role of OPN on cell migration. Furthermore, we examined the effect of secretory OPN level on cell migration by applying culture medium from DLD1-OPN stable clones or vector control (1:1 mixed with fresh culture medium) as chemoattractant for cell migration assay using DLD1 cell as model. We found that culture medium with higher OPN level significantly induced DLD1 cell migration (Fig 4C). In addition, DLD1 cell migration was significantly impaired using culture medium of siOPN transfected DLD1-OPN#1 cells when compared with that of siCTL transfected cells (Fig 4F).

In addition, our results demonstrated that overexpression of OPN in DLD1 cells significantly induced the transcription of Snail and Twist, which are inducers of epithelial-to-mesenchymal transition (EMT) process by down-regulating E-cadherin and promoting cell migration, invasion and metastasis [21–23]. As expected, the transcript and protein levels of E-cadherin were significantly reduced in DLD1-OPN stable clones (Fig 3C–3E). On the other hand, following siOPN transfection, the transcription of Snail and Twist was repressed, and resulted in elevation of E-cadherin transcription and translation (Fig 3D and 3E). These results suggested that induction of the EMT process is responsible for the enhanced cell migration of OPN-overexpressing DLD1 cells.

## Discussion

The overall prognosis of CRC patients is still unsatisfactory due to cancer metastasis, hence, it is important to develop additional biomarkers in order to enhance the prognosis of CRC patients by prediction or early detection of occult metastasis. Blood-based, minimally invasive markers would be increasing important due to the ease and convenience of sample collection. Blood CEA and CA19-9 have been commonly assessed in CRC patients, but with varying results depending on the study design and the study population [4], and their clinical association with cancer metastasis is lacking. Therefore, we believe that development of additional blood-based biomarker would be crucial for improving the prognosis of metastatic CRC patients.

Increased levels of OPN mRNA and protein have been demonstrated in CRC comparing to the non-tumor tissue [14–17]. OPN expression is regulated by a wide variety of stimuli which are associated with CRC progression and metastasis [24–27], involving complex regulatory pathways. Various growth and differentiation factors also increase OPN expression, including platelet-derived growth factor, epidermal growth factor, transforming growth factor-β, bone morphogenic proteins and inflammatory cytokines [28]. In addition, transcription of OPN is also directly activated by a number of transcriptional regulators including Wnt signaling and Tcf-4 [28], which is a well-known deregulated pathway promoting CRC. In the presence of β-catenin, Tcf-4 cooperates to stimulate OPN promoter activation and protein production [29]. We believed that the activation of these factors and signaling pathway during CRC tumor progression induces OPN expression and the subsequent cancer cell metastasis.

Plasma OPN is a potential non-invasive prognostic marker for tumor progression, invasion and metastasis in gastrointestinal cancers [30]. While high plasma OPN level has been associated with metastatic features in a variety of cancers such as gastric cancer [31], hepatocellular carcinoma, esophageal squamous cell carcinoma [32], renal cell carcinoma [33], prostate cancer [34] and breast cancer patients [35], its correlation with CRC metastasis has not been demonstrated thus far. The present study aims to examine the potential of plasma OPN levels as a diagnostic and prognostic biomarker of CRC.

This study showed that the post-operative plasma OPN level could predict the development of future metastasis in CRC patients. While most of the studies on OPN focused on the clinical significance of pre-operative plasma OPN level in cancer patients, the post-operative level has seldom been investigated. A recent HCC study which demonstrated post-operative serum OPN level could serve as surrogate serologic biomarker for monitoring treatment response and tumor recurrence [36], indicating the potential of post-operative plasma OPN as biomarker in HCC and other cancers. Our results demonstrated a significantly higher post-operative plasma OPN level in CRC patients with post-operative distant metastasis and shorter DFS, when compared with those bearing low post-operative plasma OPN level. We further showed that neither pre-operative nor post-operative CEA level was prognostic for development of post-operative metastasis, suggesting that in addition to CEA, other blood parameters (such as plasma OPN) are necessary to monitor the disease progression status of CRC patients.

Our results revealed that pre-operative plasma OPN level correlated with tumor size and distant metastasis. One possible explanation to such correlation is that plasma OPN level associates with treatment response of chemotherapy. In a non-small cell lung cancer clinical study, high pre-operative plasma OPN level was significantly associated with patient response, progression-free survival, and overall survival in patients receiving platinum-based chemotherapy [37]. In another colorectal cancer study which employed plasma OPN in their six-marker signature, they demonstrated the predictive importance of their six-marker signature for CRC patients treated with bevacizumab in combination with capecitabine/oxaliplatin [32]. Since oxaliplatin/cisplatin-based chemotherapy is widely applied to CRC patients, their findings suggest that patients bearing higher plasma OPN are more resistant to those treatments and thus more susceptible to tumor progression and distant metastasis.

Moreover, our study demonstrated that high pre-operative plasma OPN level was associated with metastasis at the time of surgical operation while high post-operative level correlated with development of distant metastasis after primary resection. The functional effects of endogenous and exogenous OPN in induction of CRC cell growth, angiogenesis, invasion and migration have been demonstrated both in *vitro* and *in vivo* [16, 38]. Our *in vitro* experiments also showed that overexpression of OPN induced cell migration in CRC cell-lines DLD1 through induction of EMT. However, no significant change was observed in the cell invasion assay, suggesting that OPN is not the unique factor necessary to stimulate invasion. Similar observation that certain factors regulate cell migration but not invasion has been reported by other cancer studies [39, 40]. DLD1 cells migrated faster when cultured in medium with higher secretory OPN level, indicating that OPN was not only a biomarker but also involved in the metastatic process. Secretory OPN is more likely to play a crucial role in liver metastasis of CRC cancer cells, since the two known receptors of OPN (integrin αv and CD44v6 proteins) are strongly expressed in hepatocytes from normal liver [41]. We believed that inhibitor targeting OPN is a potential therapeutic approach to treat CRC patients. A recent study demonstrated the efficacy of a humanized anti-OPN antibody in inhibiting breast cancer growth and metastasis *in vivo* [42], the efficacy of targeting OPN in CRC treatment by this anti-OPN antibody or other inhibitors warrants further investigations.

Plasma OPN has also been extensively investigated and shown to be a valuable biomarker in non-small-cell lung cancer (NSCLC) patients. Its level increased in early NSCLC, reduced after resection and increased with recurrence [43]. In chemotherapy-treated patients with advanced NSCLC, low plasma levels of OPN were significantly associated with improved clinical outcomes. Patients with lower levels had better response rates and higher overall and progression-free survival rates, suggesting that OPN plasma level have utility as a prognostic biomarker in chemotherapy-treated patients with unresectable NSCLC [37]. A more recent NSCLC study demonstrated that OPN level changes over time, particularly post-treatment, may yield additional prognostic information in curative-intent radiotherapy of NSCLC, which might be potentially useful in the identification of patients with high risk of death and relapse after radiotherapy. This could be beneficial in patient stratification and the decision-making process for post-radiotherapy treatment concepts. We believed that plasma OPN is a valuable biomarker in CRC. The pre-operative plasma OPN level could be applied to predict the treatment response to chemotherapy for patients with unresectable CRC, whereas the post-operative plasma OPN level could predict and monitor the response to adjuvant chemotherapy, and early detection of recurrence and metastasis. Extensive care and alternative treatment approach should be taken for CRC patients who show high or increasing plasma OPN level.

To conclude, this study demonstrated high post-operative OPN after surgery were correlated with post-operative distant metastasis, suggesting that post-operative plasma OPN level is a potential non-invasive biomarker for monitoring of CRC patients after curative resection of their primary tumor.

## Supporting Information

S1 Fig Correlation of blood OPN and CEA levels with disease-free survival of CRC patients. (A) Correlation of pre-operative plasma OPN with disease-free survival of CRC patients (p = 0.720; Log-rank test). (B) Correlation of pre-operative CEA with disease-free survival of CRC patients (p = 0.235; Log-rank test). (C) Correlation of post-operative CEA with disease-free survival of CRC patients (p = 0.410; Log-rank test). All data are representative of three independent experiments.
(TIF)

S2 Fig Functional effects of OPN on CRC cell proliferation and invasion. (A) Comparison of DLD1-OPN stable clones (DLD1-OPN#1 and #3) cell proliferation rate with that of DLD1-vector control cells (One way ANOVA). (B) Comparison of number of DLD1-OPN stable clones (DLD1-OPN#1 and #3) cells invaded with that of DLD1-vector control cells (One way ANOVA). (C) Representative photos showing the number of cells invaded. All data are representative of three independent experiments.
(TIF)

S3 Fig Original uncropped blots for Fig 3E and information of the antibodies used. (TIF)

S4 Fig Representative photos of cell migration assays. (A) Representative photos showing the number of DLD1-OPN stable clones (DLD1-OPN#1 and #3) or vector control (DLD1-Vector) migrated. (B) Representative photos showing the number of DLD1-OPN#1 cell migrated when transfected with siOPN or siOPN. (C) Representative photos showing the number of DLD1 cells migrated using culture medium from DLD1-OPN#1, DLD1-OPN#3 or vector control (1:1 mixed with fresh complete medium) as chemoattractant. (D) Representative photos showing the number of DLD1 cells migrated using culture medium from DLD1-OPN#1 transfected with siCTL or siOPN for 72 hours (1:1 mixed with fresh complete medium) as chemoattractant.
(TIF)

S1 File Supporting patient data. (XLSX)

S2 File Supporting cell-line data. (XLSX)
