# Supplementary figures and images for: Post-Operative Plasma Osteopontin Predicts Distant Metastasis in Human Colorectal Cancer
Source: PLoS One. 2015 May 11;10(5):e0126219. doi: 10.1371/journal.pone.0126219 (PMC4427310; doi:10.1371/journal.pone.0126219)

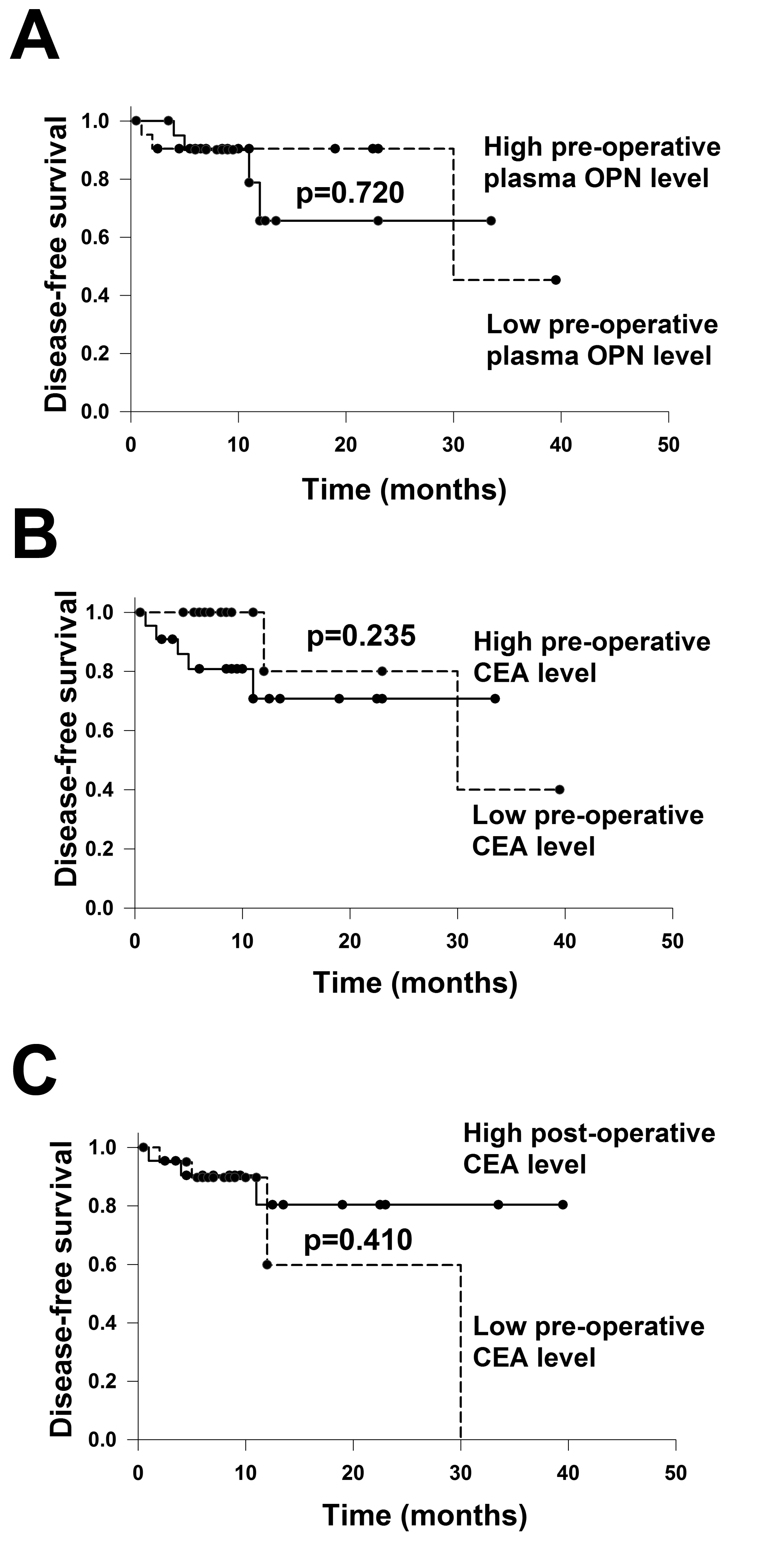

Supplement: S1 Fig — (A) Correlation of pre-operative plasma OPN with disease-free survival of CRC patients (p = 0.720; Log-rank test). (B) Correlation of pre-operative CEA with disease-free survival of CRC patients (p = 0.235; Log-rank test). (C) Correlation of post-operative CEA with disease-free survival of CRC patients (p = 0.410; Log-rank test). All data are representative of three independent experiments. (TIF) [file pone.0126219.s001.tif]

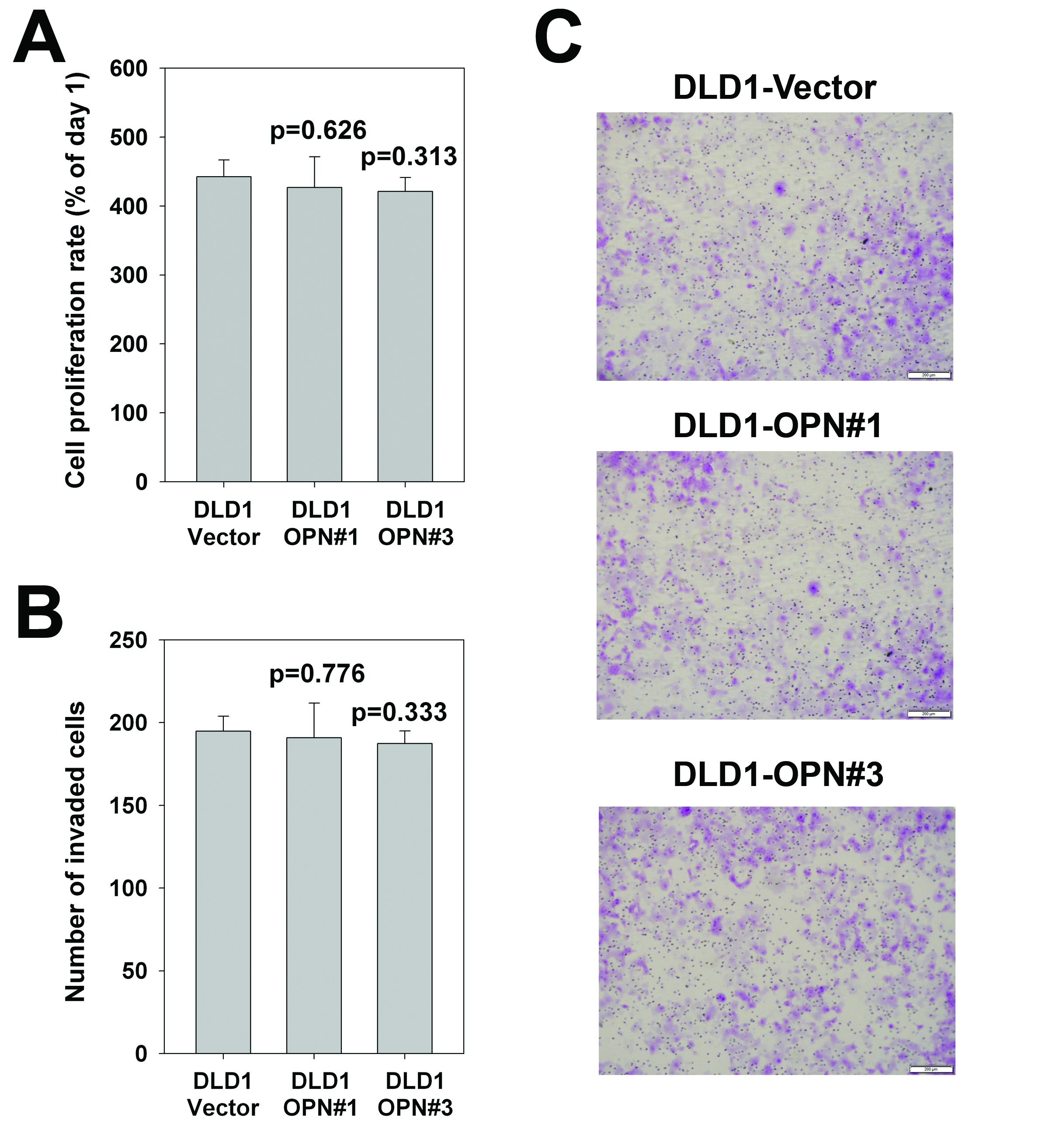

Supplement: S2 Fig — (A) Comparison of DLD1-OPN stable clones (DLD1-OPN#1 and #3) cell proliferation rate with that of DLD1-vector control cells (One way ANOVA). (B) Comparison of number of DLD1-OPN stable clones (DLD1-OPN#1 and #3) cells invaded with that of DLD1-vector control cells (One way ANOVA). (C) Representative photos showing the number of cells invaded. All data are representative of three independent experiments. (TIF) [file pone.0126219.s002.tif]

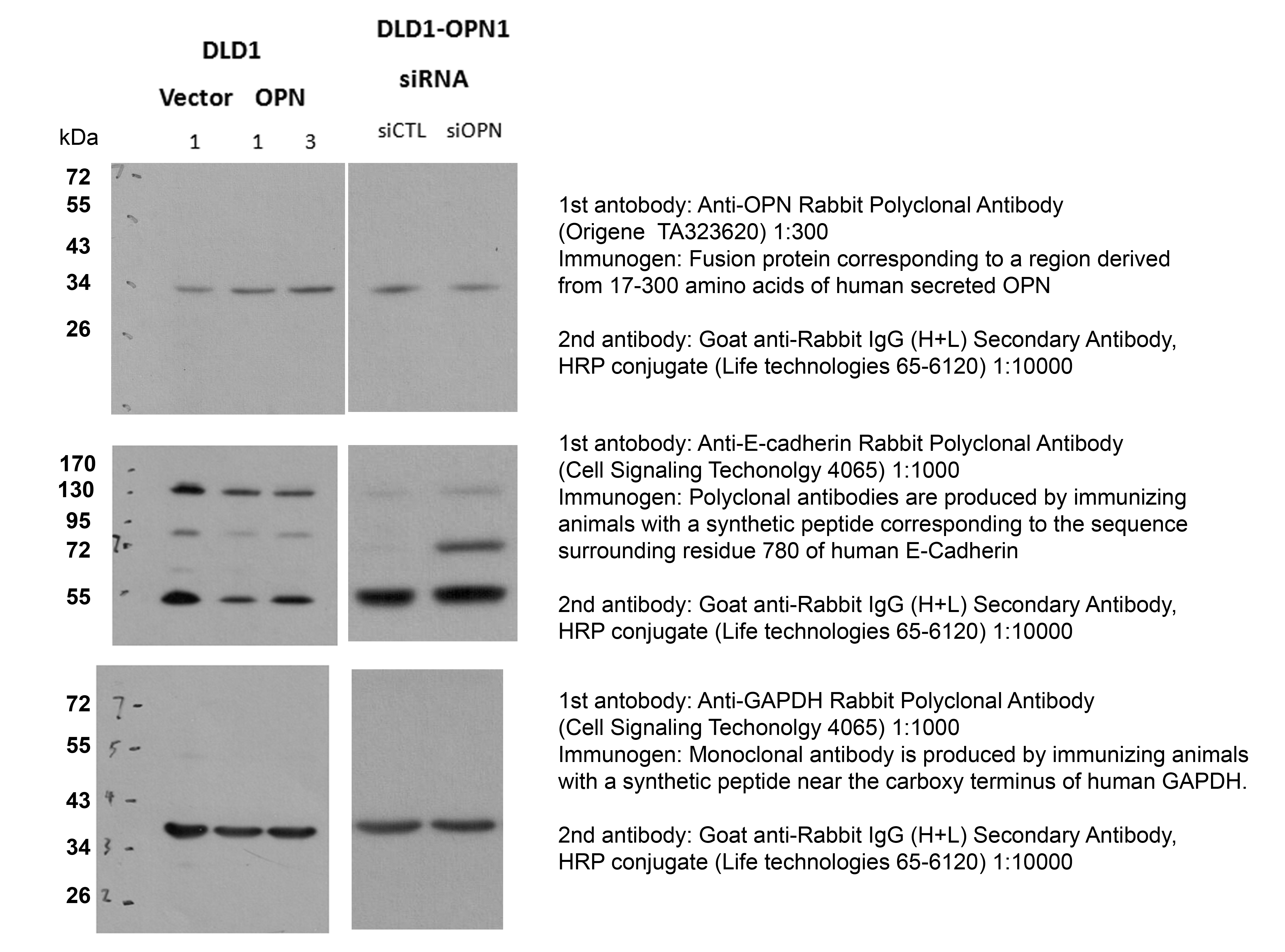

Supplement: S3 Fig — (TIF) [file pone.0126219.s003.tif]

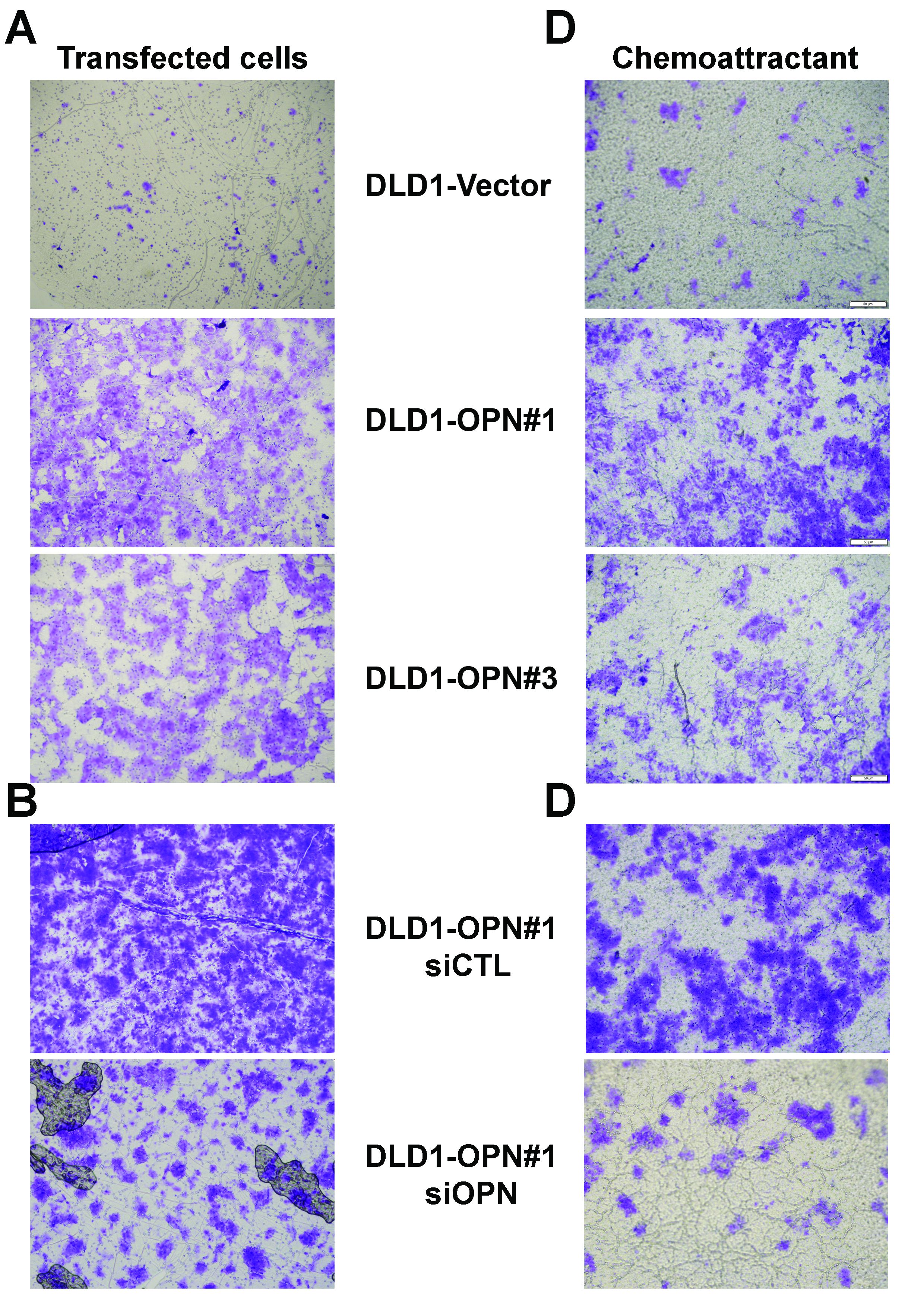

Supplement: S4 Fig — (A) Representative photos showing the number of DLD1-OPN stable clones (DLD1-OPN#1 and #3) or vector control (DLD1-Vector) migrated. (B) Representative photos showing the number of DLD1-OPN#1 cell migrated when transfected with siOPN or siOPN. (C) Representative photos showing the number of DLD1 cells migrated using culture medium from DLD1-OPN#1, DLD1-OPN#3 or vector control (1:1 mixed with fresh complete medium) as chemoattractant. (D) Representative photos showing the number of DLD1 cells migrated using culture medium from DLD1-OPN#1 transfected with siCTL or siOPN for 72 hours (1:1 mixed with fresh complete medium) as chemoattractant. (TIF) [file pone.0126219.s004.tif]
